# Myeloid-derived suppressor cells: mechanisms of action and recent advances in their role in transplant tolerance

**DOI:** 10.3389/fimmu.2012.00208

**Published:** 2012-07-17

**Authors:** Nahzli Dilek, Romain Vuillefroy de Silly, Gilles Blancho, Bernard Vanhove

**Affiliations:** ^1^ INSERM, UMR-S 1064,Nantes, France; ^2^ Effimune S.A.S,Nantes, France; ^3^ Faculté de Médecine, Université de Nantes,Nantes, France; ^4^ Institute of Transplantation - Urology - Nephrology, University Hospital of Nantes (Academia),INSERM Unit 643, Nantes, France

**Keywords:** immune suppression, myeloid suppressor cells, tolerance, transplantation

## Abstract

Myeloid-derived suppressor cells (MDSC) are a heterogeneous population of immature hematopoietic precursors known to suppress immune responses in infection, chronic inflammation, cancer, and autoimmunity. In this paper, we review recent findings detailing their mode of action and discuss recent reports that suggest that MDSC are also expanded during transplantation and that modulation of MDSC can participate in preventing graft rejection as well as graft-versus-host disease.

## INTRODUCTION

In the 1980s, a new cell population known as natural suppressor cells, distinct from T and NK cells, was described in tumor-bearing mice (Strober, 1984; Maier et al., 1989). Generated in bone marrow under the influence of soluble factors produced by tumors, these cells derive from a mixed and heterogeneous population of myeloid cells found at different differentiation stages. They have been defined as myeloid suppressive cells because of their ability to suppress immune responses (Bronte et al., 1998, 1999, 2000). To minimize the confusion with existing mesenchymal stem cells, Gabrilovich (2007) proposed to name these cells “myeloid-derived suppressor cells” (MDSC). In mice, MDSC accumulate in the lymphatic organs (Ezernitchi et al., 2006) after the development of various diseases such as infections (Marshall et al., 2001; Goni et al., 2002; Mencacci et al., 2002), chronic inflammation, tumor growth, graft-versus-host disease (GVHD; Bobe et al., 1999) and immune stress due to superantigen stimulation (staphylococcal endotoxin A, SEA; Cauley et al., 2000). In mice, MDSC are characterized by the expression of myeloid cell markers, such as GR-1 (Ly6G and Ly6C) and CD11b (Bronte et al., 1998), as well as immature cell markers, such as CD31 (Bronte et al., 2000). Two subsets of MDSC were also described: monocytic MDSC, which have CD11b^+^Ly6G^-^Ly6C^High^ phenotype, and granulocytic MDSC, which have CD11b^+^Ly6G^+^Ly6C^+^^/^^-^ phenotype (Movahedi et al., 2008; Youn et al., 2008). Other markers correlated to their suppressive function have been identified as CD80 (Mencacci et al., 2002), CD115 (Huang et al., 2006), or CD16 (Marshall et al., 2001). They also express MHC class I molecules, but not MHC class II molecules (Gabrilovich et al., 2001). In humans, MDSC accumulate in cancer patients (Pak et al., 1995; Almand et al., 2001) and are defined by the expression of immature markers such as CD34, CD33, CD15, and CD16. Moreover, CD14^+^HLA-DR^-^^/low^ MDSC have been recently characterized in cancer patients (Hoechst et al., 2008), suggesting that as is the case with mice, various human tumors induce different MDSC subsets. In the presence of appropriate growth factors [IL-4 + granulocyte macrophage colony-stimulating factor (GM-CSF) or TNF-α + GM-CSF], MDSC can differentiate into efficient antigen-presenting cells (APC), either DC or macrophages by increasing the expression of costimulatory molecules and MHC class II molecules (Bronte et al., 2000; Li et al., 2004).

## CONTROL OF MDSC BY CYTOKINES

**FIGURE 1:**
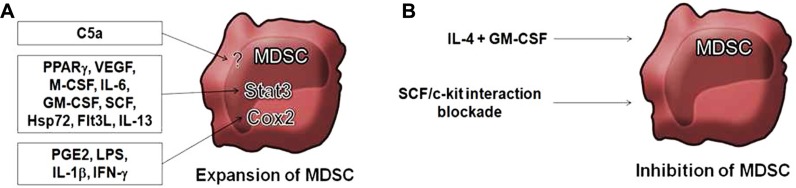
**Mechanisms of suppression by MDSC.** **(A)** Arg1, arginase-1. Arg1 induces arginine deprivation. iNOS, inducible nitric oxide synthase. iNOS induces nitric oxide (NO) production (that can be derived into reactive nitrogen species, RNS). Arg1 activity leads to CD3ζ down-modulation (Rodriguez et al., 2007; Highfill et al., 2010), TCR CD3ζ nitrosylation (Nagaraj et al., 2007, 2010), and natural Treg (nTreg) expansion (Serafini et al., 2006, 2008), while iNOS activity leads to T cell apoptosis (Brito et al., 1999; Jia et al., 2010) and inhibition of T cell proliferation (Rodriguez et al., 2007; Cripps et al., 2010). **(B)** eNOS, endothelial nitric oxide synthase. NOX2, NADPH oxidase 2. The enzyme induces reactive oxygen species (ROS) production and, together with eNOS and/or iNOS activities, can induce RNS production. NOX2 leads to inhibition of T cell proliferation through ROS production (Tacke and Kurts, 2011), TCR CD3ζ nitration (Nagaraj et al., 2010) and MHC class I nitration (Lu et al., 2011). **(C)** HO-1, heme oxygenase 1. The enzyme leads to inhibition of T cell proliferation probably through CO production (De Wilde et al., 2009). **(D)** Cys, cysteine. Cys_2_, cystine. GSH, glutathione. MDSC compete with dendritic cells (DCs) for Cys_2_ import from the extracellular milieu. This prevents DCs from providing sufficient Cys to T cells for GSH production, thus inhibiting T cell proliferation (Srivastava et al., 2010). Dotted arrows show physiological import/export inhibited by MDSC activity. **(E)** ADAM17, ADAM metallopeptidase domain 17. ADAM17 activity leads to cleavage of L-selectin (CD62L) ectodomain resulting in inhibition of the homing to lymph nodes and sites of inflammation (Hanson et al., 2009). **(F)** Membrane-bound TGF-β1 leads to NK cell anergy, resulting in inhibition of NKG2D and IFN-γ expression (Li et al., 2009). TGF-β production leads to inhibition of cytotoxic T lymphocytes (CTL; Terabe et al., 2003). In an IFN-γ rich environment, TGF-β plus IL-10 lead to expansion of induced-Treg (iTreg; Huang et al., 2006). IL-10 production promotes Th2 deviation and macrophage type 2 (Mφ2) polarization that secrete lower amounts of IL-12 and higher amounts of IL-10 (Sinha et al., 2007b). Question marks denote suggested, but unproven, participations.

Many studies have shown that inflammatory environments induce the production and the accumulation of MDSC able to block CD4 and CD8- immune responses and lead to cancer development. Indeed, tumor cells secrete a large variety of cytokines that allow the recruitment of MDSC in lymphoid organs or peripheral blood and direct their differentiation into suppressor cells (Kusmartsev et al., 2003). That global inflammation controls MDSC recruitment is best illustrated by observations showing that the reduction of inflammatory potential in IL-1R^-^^/^^-^ mice allows delaying MDSC accumulation and then reducing tumor and metastatic growth (Bunt et al., 2007; **Figure 1**). One key factor controlling MDSC expansion and the development of cancer is peroxisome proliferator-activated receptor-gamma (PPARγ; Wu et al., 2012). Also vascular endothelial growth factor (VEGF; Melani et al., 2003), macrophage colony-stimulating factor (M-CSF; Kusmartsev et al., 2003) or IL-6 (Bunt et al., 2007) are required for MDSC expansion (Ohm and Carbone, 2001). Indeed, they prevent MDSC differentiation into mature DC through a mechanism involving the activation of STAT3 signaling pathway (Gabrilovich, 2004; Nefedova et al., 2004). By contrast, in a mouse cancer model, the use of siRNA blocking expression of stem cell factor (SCF) or blockade of SCF/c-kit receptor interaction allowed to reduce MDSC expansion and restore T lymphocyte proliferation, thus resulting in tumor rejection (Pan et al., 2008). GM-CSF also induces MDSC expansion which suppresses tumor-specific CD8^+^ T cell response. However, in combination with IL-4, GM-CSF induces MDSC differentiation into mature DC capable to activate immune responses (Bronte et al., 1999; Mellstedt et al., 1999). PGE2 also, as well as other COX2 activators as lipopolysaccharide, IL-1β, and IFN-γ, by inducing expression of COX2 in monocytes, blocks their differentiation into mature DCs and induces a typical MDSC phenotype (Sinha et al., 2007a; Obermajer et al., 2011). In addition IFN-γ produced by T cells in tumor-bearing mice was shown to make MDSC responsive to IL-13 and suppressive (Gallina et al., 2006). Another important factor is Hsp72 that was shown essential for expansion, activation, and suppressive function of murine and human MDSC, also through STAT3 signaling pathway (Chalmin et al., 2010). Another study demonstrated that injection of fms-like tyrosine kinase 3 ligand (Flt3L) encoding adenoviruses in tumor-bearing mice resulted in the increase of spleen DC, T, B lymphocytes and NK cells but also of MDSC which dominated and blocked anti-tumor activity of effector cells (Solheim et al., 2007). Finally, it was recently shown that the complement anaphylatoxin C5a increases tumor infiltrating MDCS and gives them a suppressive activity through reactive oxygen species (ROS) and reactive nitrogen species (RNS) regulation (Markiewski et al., 2008). Several tumor-derived factors such as TGF-β, IL-3, IL-6, IL-10, platelet-derived growth factors, and GM-CSF could also induce ROS production by MDSC (Sauer et al., 2001). Beside soluble factors, MDSC are controlled by their expression of Fas which leads to cell apoptosis after contact with Fas-L positive activated T cells (Sinha et al., 2011).

## MECHANISMS OF SUPPRESSION

**FIGURE 2:**
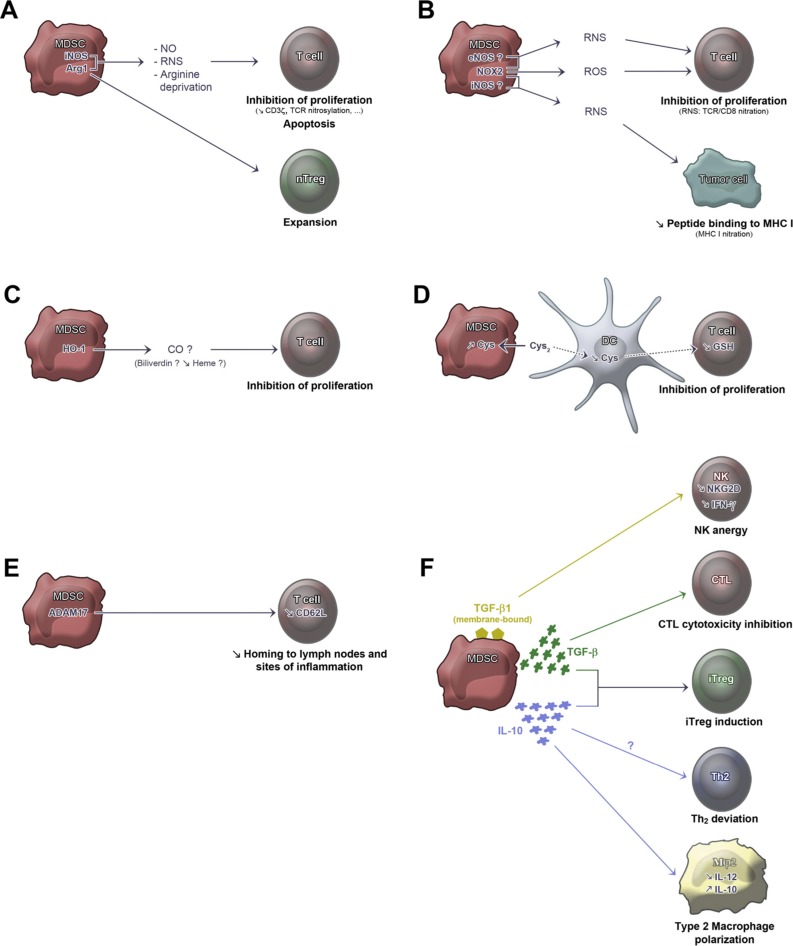
**Control of MDSC by cytokines.** **(A)** Inflammatory environments lead to expansion of MDSC by activation of the STAT3 signaling pathway by several factors including granulocyte macrophage colony-stimulating factor (GM-CSF; Bronte et al., 1999); macrophage colony-stimulating factor (M-CSF; Kusmartsev et al., 2003); IL-6 (Bunt et al., 2007); peroxisome proliferator-activated receptor-gamma (PPARγ; Wu et al., 2012); vascular endothelial growth factor (VEGF; Ohm and Carbone, 2001; Melani et al., 2003); stem cell factor (SCF; Mellstedt et al., 1999); IL-13 (Gallina et al., 2006); Hps72 (Chalmin et al., 2010); and fms-like tyrosine kinase 3 ligand (Flt3L; Solheim et al., 2007). Agonists of the COX2 pathway also result in expansion of MDSC, including prostaglandin E2 (PGE2), lipopolysaccharide (LPS), IL-1β, and IFN-γ (Obermajer et al., 2011). The complement anaphylatoxin C5a is also described to induce MDSC (Markiewski et al., 2008). **(B)** Blockade of SCF/c-kit interaction or SCF blockade by siRNA reduce MDSC expansion (Mellstedt et al., 1999). The combination of IL-4 and GM-CSF inhibits MDSC function by inducing their differentiation into mature DC (Bronte et al., 1999; Sinha et al., 2007a).

Several regulatory mechanisms have been associated to MDSC and new ones are being uncovered (summarized in **Figure 2**), a phenomenon probably due to their heterogeneity. Following an immune stress due to GM-CSF production by tumor cells, MDSC accumulate in lymphoid organs where they suppress proliferation of and cytokine production by T and B cells activated by alloantigens (Schmidt-Wolf et al., 1992) or by CD3 stimulation (Young et al., 1996). Indeed, MDSC block the cell cycle at the G0/G1 phases in a contact-dependent manner (Gabrilovich, 2004; Kusmartsev et al., 2004). The suppressive activity of MDSC also depends on the release of IFN-γ by target T cells (Mazzoni et al., 2002). MDSC can also inhibit NK cell activity through membrane-bound TGF-β1, resulting in inhibition of IFN-γ and NKG2D expression (Li et al., 2009). The effect shows a high efficacy since addition *in vitro* of only 3% of MDSC was able to completely block T cell proliferation (Mazzoni et al., 2002). To control T cell response and in response to signals provided by activated T cells, activated MDSC use two enzymes involved in L-arginine metabolism: iNOS which allows NO generation (Kusmartsev et al., 2000) and arginase 1 (Arg1) which depletes arginine from the environment (Mills et al., 1992; Bronte and Zanovello, 2005; Gallina et al., 2006). These two mechanisms of action appear to be used by monocytic and granulocytic subtypes of MDSC, respectively (Movahedi et al., 2008). *In vitro*, iNOS inhibitors (L-NMMA) combined or not with Arg1 inhibitors (Kusmartsev et al., 2000; Bronte et al., 2005) block inhibition of T cells by MDSC. Similarly, phosphodiesterase-5 inhibitors delay tumor progression by decreasing Arg1 and iNOS expression and by regulating the suppressive machinery of MDSC. The activation of either of these enzymes inhibits T cell proliferation by interfering with the transduction of intracellular signals and by inducing T cell apoptosis (Brito et al., 1999; Bronte et al., 2003). In fact, the loss of L-arginine inhibits T cell proliferation through several mechanisms such as the decrease of CD3ζ chain expression and the inhibition of Cyclin D3**and *Cyclin*-*dependent Kinase* (cdk)-4 upregulation (Rodriguez et al., 2002, 2004, 2007; Highfill et al., 2010). Interestingly, arginine deprivation of T cells can reproduce the activity of MDSC by blocking the cell cycle at the G0/G1 stage (Rodriguez et al., 2002). Regulation of L-arginine concentration in the microenvironment is therefore an important mechanism to modulate CD3ζ chain expression of T cell receptor (TCR) and T cell function. Another important consequence of Arg1 activity is the induction of expansion of natural T regulatory cells (nTreg; Serafini et al., 2008). The second mechanism of action involving iNOS and NO production suppresses T cell function through other mechanisms involving the inhibition of JAK3 and STAT5, a mechanism shared with suppressive macrophages (Bingisser et al., 1998), the inhibition of MHC class II expression (Harari and Liao, 2004) and the induction of T cell apoptosis (Rivoltini et al., 2002; Jia et al., 2010). De Wilde et al. (2009) showed for the first time that another enzyme, heme oxygenase 1 (HO-1), is also associated with suppressive function of MDSC. Indeed, endotoxin-induced MDSC produce IL-10 and express HO-1, an enzyme involved in the response to oxidative stress and featuring immunomodulatory and cytoprotective properties. Specific HO-1 inhibition by tin protoporphyrin completely canceled suppression and IL-10 production by MDSC, showing the important role of this enzyme in MDSC function.

In addition to their direct suppressive action, MDSC may also have an indirect action on the inhibition of T lymphocyte proliferation by promoting the development of inducible CD4^+^CD25^+^Foxp3^+^ T regulatory cells (iTreg; Huang et al., 2006). The development of these Treg is independent from “classical” MDSC suppressive mechanisms involving arginine metabolism, but is linked to IL-10 plus TGF-β production. Moreover, preventing CD80 expression on MDSC or the use of anti-CTLA-4 antibodies delay tumor growth, suggesting that CTLA-4/CD80 interaction between MDSC and Treg is necessary for their activity or their development (Yang et al., 2006). Another study analyzed the interaction of MDSC with macrophages in a mouse cancer model and showed that, through IL-10 secretion, MDSC induced a type-2 polarization of macrophages which is characterized by a decrease of IL-12 secretion and that promotes tumor growth (Sinha et al., 2007b). IL-10 secretion by MDSC might also account for the Th2 deviation associated with MDSC activity (Sinha et al., 2007b). In addition, cytotoxic T lymphocytes (CTL) cytotoxicity can be prevented by MDSC through TGF-β production (Terabe et al., 2003).

More recently, RNS, and particularly peroxynitrites, emerged as a key mediator of T cell function suppression by MDSC. Indeed, peroxynitrites are a product of a chemical reaction between NO and superoxide anion, and is one of the most powerful oxidizers. It induces amino acid nitration and nitrosylation such as cysteine, methionine, tryptophan, and tyrosine (Vickers et al., 1999). High levels of peroxynitrites have been found in areas where inflammatory cells and MDSC accumulate. These high levels of peroxynitrites have been also associated with tumor progression in many types of cancer (Vickers et al., 1999; Schmielau and Finn, 2001; Mantovani et al., 2003; Szuster-Ciesielska et al., 2004; Kusmartsev et al., 2005; Nagaraj et al., 2007) which have been linked to the absence of T cell responses. One study indeed reported the infiltration of differentiated but inactivated CD8^+^ T cells in prostate adenocarcinoma in human (Bronte et al., 2005). It appears that the peroxynitrite production by MDSC during direct contacts with T cells leads to TCR and CD8 molecule nitration, changing the specific binding peptide of T cells and making them intensive to specific antigen stimulation (Nagaraj et al., 2007). Also, it has been shown that MDSC are able to induce TCR/CD3ζ complex disruption through tyrosine nitrosylation/nitration, partly through NADPH oxidase 2 (NOX2) activity (Nagaraj et al., 2010). This might explain some conflicting results showing T cell function defects without modification of CD3ζ expression, especially since CD3ζ might be degraded later on (Levey and Srivastava, 1995). Further, in tumor cells peptide binding to MHC class I can be prevented by MDSC-induced MHC nitration through RNS production in a NOX2-dependent manner (Lu et al., 2011). Another important factor that contributes to suppressive activity of MDSC is the production of ROS. The increase production of ROS has emerged as one of the main features of MDSC in tumor-bearing mice and cancer patients (Bronte et al., 2000; Schmielau and Finn, 2001; Mantovani et al., 2003; Szuster-Ciesielska et al., 2004; Kusmartsev et al., 2005; Agostinelli and Seiler, 2006; Youn et al., 2008), partly through NOX2 activity (Corzo et al., 2009). *In vitro* inhibition of ROS production by MDSC derived from these mice and patients completely cancels the suppressive effect of these cells (Bronte et al., 2000; Schmielau and Finn, 2001; Szuster-Ciesielska et al., 2004).

Two other mechanisms of suppression have been recently identified. First, by expressing ADAM metallopeptidase domain 17 (ADAM17), MDSC induce the cleavage of L-selectin (CD62L) ectodomain on T cells, a membrane molecule involved in the migration of naïve T cells into lymph nodes. Thus, CD4 and CD8 cells become unable to migrate into lymph nodes or inflammatory sites where they are supposed to be activated (Hanson et al., 2009). Finally, two studies identified a new mechanism of suppression based on modulation of local amino acid metabolism and homeostasis. This mechanism, shared with FoxP3^+^ Treg is called cysteine/cystine deprivation (Yan et al., 2009, 2010). Some time ago, it has been described that mammalian cells can obtain cysteine through three main pathways (Bannai, 1984). Foremost, they can metabolize cysteine from methionine through transsulfuration, a pathway catalyzed by cystathionase, a pyridoxal phosphate dependent rate-limiting enzyme. Cells can also import cystine (the oxidized form of cysteine) from the extracellular environment through the Xc^-^ transporter that also exports glutamate at the same time. Alternatively cells can import cysteine from the extracellular environment through the alanine–serine–cysteine (ASC) neutral amino acid transporter (that can also export cysteine). However, the ASC pathway is limited by the fact that cysteine in the medium or in plasma, is predominantly present under its oxidized form, cystine, which cannot use the ASC transporter. Cysteine is a non-essential amino acid because it can be produced through the transsulfuration pathway. However its production is vital considering this is the limiting precursor in the production of the tripeptide glutathione, the major intracellular antioxidant molecule. In order to proliferate, T cells need to produce glutathione in a sufficient manner and thus need to replenish cysteine content to allow glutathione turnover (Suthanthiran et al., 1990). They do express the ASC neutral amino acid transporter but are deficient in cystathionase and Xc^-^ transporter. Of interest, Angelini et al. (2002) showed that after APC–T cell interaction, APC allows the conversion of cystine into cysteine in the medium, thereby providing cysteine in the reduced form to T cells in order to proliferate. This is, in part, due to a process involving APC import of cystine from the medium by the Xc^-^ transporter, followed by its intracellular reduction (i.e., the redox potential being highly reduced inside cells) and by subsequent export of cysteine through the ASC transporter. The model therefore presents APC as “feeder cells” for T cells, delivering cysteine that otherwise would be lacking for T cell proliferation. Recently, Srivastava et al. (2010) studied mouse MDSC in a tumor context. They showed that MDSC express the Xc^-^ transporter, but lack the cystathionase enzyme and the ASC transporter. Thus, MDSC seem to possess the same capacities as APC to import cystine, but are unable to export cysteine and can therefore be considered as “cystine/cysteine sinks.” Interestingly, by adding a donor of cysteine, or a reducing agent (i.e., β-mercaptoethanol), that allows conversion of cystine to cysteine in the medium, the MDSC-induced T cells suppression was partially prevented, suggesting indeed that MDSC inhibit T cell proliferation, in part, by depleting the environment of cysteine (Srivastava et al., 2010). Consistent with these results, by co culturing APC with MDSC, Srivastava et al. (2010) observed reduced levels of extracellular cysteine contents as compared to APC alone. All these results argue for a new mechanism of suppression involving cysteine homeostasis: MDSC may import cystine from the medium and induce cystine starvation in the microenvironment (since they do not export it), thus preventing APC from providing sufficient cysteine for T cells proliferation.

## MDSC AND TRANSPLANTATION

In transplantation, in contrast with Treg, the role of MDSC is not well characterized. It was first described in a renal allograft tolerance induction model in rats. In this model, tolerance was induced by selective costimulation blockade (Dugast et al., 2008). An accumulation of CD3^-^ClassII^-^CD11b^+^CD80/86^+^ cells was observed in the blood of tolerant recipients and cells with a similar phenotype were also detected into the tolerated graft. These cells identified as MDSC inhibited proliferation of effector T cells and induced a contact-dependent apoptosis in an iNOS-dependent manner. The importance of iNOS was highlighted by the observation that administration of iNOS inhibitors induced rejection of tolerated allograft. Another study showed that SHIP (inositol polyphosphate-5-phosphatase) deficient mice were able to accept an allogeneic bone marrow transplant without developing GVHD. SHIP is involved in the regulation of cell survival, proliferation, and differentiation of myeloid cells as well as in the regulation of MDSC homeostasis (Liu et al., 1999). Thereby, the inhibition of GVHD in these SHIP^-^^/^^-^ mice appears to be due to accumulation of MDSC which suppress allogeneic T cell responses (Ghansah et al., 2004; Paraiso et al., 2007). Also in mice, adoptive transfer of functional MDSC generated *in vitro* from murine embryonic stem cells (ES) prevented GVHD via IL-10 and iNOS and was able to induce the development of CD4^+^CD25^+^Foxp3^+^ Treg (Zhou et al., 2010). Likewise, Highfill et al. (2010) showed that bone marrow-derived MDSC inhibited GVHD by an Arg1 dependent mechanism, which itself is regulated by IL-13. There has also been evidence that MDSC use the HO-1 to suppress alloreactivity (De Wilde et al., 2009). In another mouse skin graft model, the *in vivo* induction of Gr-1^+^CD11b^+^ MDSC by Neupogen, the recombinant human granulocyte colony-stimulating factor (rhG-CSF) or the induction of CD4^+^Foxp3^+^ Treg by IL-2 complexes (IL-2C) similarly prolonged allograft survival (Adeegbe et al., 2010). Interestingly, when animals were treated with a combination of IL-2C and Neupogen, a further increase of Treg was observed. This observation suggested a possible cooperation between MDSC and Treg to promote allograft survival. Such a MDSC–Treg cooperation had also been studied *in vitro*: it was shown that MDSC interaction with activated effector T cells resulted in the upregulation of iNOS and in the activation of the suppressive action whereas interaction with activated Treg cells failed to upregulate iNOS. As a result MDSC could block effector T cell proliferation but could not block proliferation of Treg cells (Dugast et al., 2008). However, molecular interactions driving this differential suppression on T effector and T regulatory cells have not been elucidated.

Another mechanism of action of MDSC uncovered in the context of transplantation involves the inhibitory receptors Ig-like transcript 2 (ILT2), an inhibitory TCR whose activation causes a decrease of T cell activation. In a model of skin allograft in mice, ILT2 interaction with HLA-G was shown to induce expansion of a MDSC population with a significant suppressive activity (Zhang et al., 2008). In addition, survival of skin allografts was prolonged after adoptive transfer of MDSC from ILT2 transgenic mice. In that case, MDSC accumulated into the graft. MDSC expansion resulting from HLA-G/ILT2 interaction appeared to induce VEGF and GM-CSF. ILT2 transgenic mice also have an increased expression of Arg1, probably due to IL-4 and IL-13 over-expression in MDSC (Zhang et al., 2008).

MDSC can modulate rejection after pancreatic islets allografts in diabetic mice (Marigo et al., 2010). Indeed adoptive transfer of MDSC derived from bone marrow and generated by GM-CSF and IL-6 increases significantly the percentage of long-term survival mice transplanted with allogeneic islets in the absence of immunosuppression. Tolerance was achieved by inhibition of IFN-γ producing T cells and was found dependent on the expression by myeloid cells of regulatory transcription factor CCAAT/enhancer binding protein beta (C/EBPβ), a downstream target of Ras signaling involved in positive and negative cell cycle regulation. Finally, in a mouse tolerance model of heart transplantation, the group of Ochando showed increased numbers of CD11b^+^CD115^+^Gr1^+^ monocytic MDSC. Shortly after transplantation they migrated from the bone marrow to the transplant where they participated in the induction of Treg and prevented initiation of adaptive immune responses (Garcia et al., 2010). Lastly, elevated frequencies of circulating CD14^Neg^ and CD14^Pos^ MDSC have recently been recorded in patients recipients of renal transplants and CD14^Neg^ MDSC were found associated with occurrence of squamous cell carcinoma in these patients (Hock et al., 2012). Thus MDSC has potential functional relevance in kidney graft recipients with respect to transplant tolerance but also cancer immunosurveillance. The reported involvement of MDSC in transplantation is summarized in **Table 1**.

**Table 1:** Reported involvement of MDSC in transplantation.

Phenotype	Species	Models	Mechanisms	Reference
CD3^-^ClassII^-^CD11b^+^CD80/86^+^	Rat	Renal transplant tolerance	Accumulation; iNOS	Dugast et al. (2008)
Gr-1^+^CD11b^+^	Mouse	GVHD inhibition	Altered Ag processing by DC	Ghansah et al. (2004), Paraiso et al. (2007)
CD115^+^Gr-1^+^F4/80^+^	Mouse	GVHD prevention	IL-10; iNOS	Zhou et al. (2010)
CD11b^+^Ly6G^low^Ly6C^+^	Mouse	GVHD inhibition	Arg1	Highfill et al. (2010)
Gr-1^+^CD11b^+^	Mouse	Skin allograft; long-term survival	iNOS	Adeegbe et al. (2010)
Gr-1^+^CD11b^+^	Mouse	Skin allograft; long-term survival	Arg1	Zhang et al. (2008)
Gr-1^+^CD11b^+^ IL-4Rα^+^	Mouse	Islet allograft tolerance	C/EBPβ factor; Arg1; iNOS	Marigo et al. (2010)
Gr-1^+^CD115^+^CD11b^+^	Mouse	Cardiac transplant tolerance	IFN-γ-dependent pathways	Garcia et al. (2010)
CD33^+^HLA-DR^-^CD11b^+^CD14^+/-^	Human	Renal transplantation	Accumulation	Hock et al. (2012)

In conclusion, probably due to their heterogeneous origin, MDSC use several suppressive mechanisms which enable them to control adaptive immune responses. In addition to their recognized role in tumor tolerance, they potentially exert a role in the induction and maintenance of transplant tolerance. However, whether MDSC generated post-transplantation result from creeping inflammation and interferes with immunosurveillance or potentially constitute an appropriate immune regulatory response, as recently explored (Hock et al., 2012), remains to be established. Further phenotyping MDSC post-transplantation in humans might help deciphering their potential “physiological” role and understanding whether, in spite of their non-specific immunosuppressive activity, they might be used in cell therapies in synergy with existing immunosuppressive therapies.

## Conflict of Interest Statement

The authors declare that the research was conducted in the absence of any commercial or financial relationships that could be construed as a potential conflict of interest.
